# Teacher Efficacy, Collective Self-Esteem, and Organizational Commitment of Childcare Teachers: A Moderated Mediation Model of Social Support

**DOI:** 10.3389/fpsyg.2019.00955

**Published:** 2019-05-21

**Authors:** Myung-Sun Chung

**Affiliations:** Department of Counseling Psychology Education, Joongbu University, Goyang-si, South Korea

**Keywords:** social support, teacher efficacy, collective self-esteem, organizational commitment, moderated mediation

## Abstract

This study explored the moderated mediation model from the viewpoint of whether the job resource of social support can reinforce relationships among teacher efficacy, collective self-esteem, and organizational commitment based on the Job Demands-Resources (JD-R) model. A sample of 212 childcare teachers completed a self-report battery that included measures of teacher efficacy, collective self-esteem, organizational commitment, and social support. The results indicated that collective self-esteem partially mediated the relationship between teacher efficacy and organizational commitment. The findings also demonstrated that teacher efficacy translated into a higher organizational commitment among teachers that perceive relatively higher degrees of social support in both the indirect effect through collective self-esteem and the direct effect without collective self-esteem. The study highlights the need to establish public childcare policies that are conducive to increasing social support for teachers. Based on these results, the benefits of understanding this moderated mediation path, the limitations of the study, and future research directions are discussed.

## Introduction

The nursery school, an institution that oversees the most fundamental education among common education institutions, is regarded as a basic educational service system in South Korea (hereafter “Korea”). However, nursery schools were the last form of education to be defined as compulsory education among Korean public education institutions. In addition, unlike elementary, middle, or high school teachers, childcare teachers exhibit high turnover rates, and it is one of the thirty occupational categories among 10,971 occupations, excluding similar occupation names that involve a significant emotional investment (Korea Research Institute for Vocational Education and Training, 2013). Therefore, recently, an emphasis has been placed on organizational commitment in Korea from the perspective of improving the organizational efficiency and human resource management of teachers. The concept of organizational commitment describes an individual as being positively involved with their organization, and it is a type of psychological attachment that binds an individual to a common goal (Mowday et al., 1979; Meyer and Herscovitch, 2001; Robbins, 2001). According to the Three-Component Model (TCM), organizational commitment encompasses affective, continuance, and normative commitments (Meyer and Allen, 1991; Dunham et al., 1994). From a teacher’s perspective, organizational commitment is the psychological connectedness to the school organization, the willingness to remain in the educational institution from a sense of unity, and the acceptance of the goals and values pursued by that organization (Ma, 2004; Chung, 2015).

Many researchers have proposed that teacher efficacy is one of the more vital qualities required of teachers for organizational commitment (Kushman, 1992; Bogler and Somech, 2004; Kim and Chung, 2014). Teacher efficacy refers to teachers’ perceptions of their influence on a student’s learning and the beliefs about and expectations of their teaching abilities and behavior (Ashton, 1985; Riggs and Enochs, 1990). As the saying makes clear, “The quality of education cannot outdo the quality of teachers.” Teacher efficacy is the solution to many problems within the educational field and is, therefore, an important requirement. In particular, childcare teachers take on many duties, including daily childcare activities, teaching, and classroom management, as well as more sophisticated responsibilities such as diagnosis, decision making, and judgment (Broudy, 1984; Kim and Chung, 2014; Lin and Magnuson, 2018). As childcare teachers are professionals concerned with a child’s most formative period, teacher efficacy is vital because efficient and confident educational commitment is difficult without it (Kim and Chung, 2014). In terms of educational effectiveness and efficiency, teacher efficacy facilitates individual teachers’ attachment and commitment to an educational organization (Moore and Esselman, 1992; Ross, 1992). A teacher’s passion, morale, and motivation can serve as the starting point for organizational commitment (Tschannen-Moran et al., 1998; Tschannen-Moran and Hoy, 2001; Kim and Kim, 2004), and teachers with high efficacy adjust their teaching methods effectively according to the situation (Saka et al., 2016), believe they can influence students regardless of their backgrounds, and commit to the goals of their childcare institution with enthusiasm (Gibson and Dembo, 1984; Kim and Chung, 2014). In addition, teacher efficacy prevents burnout by helping teachers perceive various educational scenarios as less threatening (Abbey and Esposito, 1985). Korean teachers have participated in various policy-based programs to improve teacher efficacy, and these programs have ultimately served as a form of human resource management affecting organizational commitment.

In contrast, the efficacy possessed by some individual teachers leads to perceived presence and esteem for the affiliated group, contributing to a sense of pride for the teaching group or educational institution (Lee and Lee, 2012). This can be expressed as collective self-esteem (CSES)—a more comprehensive and complete concept than collective efficacy, which is the aggregation of personal teacher efficacy (Goddard, 2001). CSES refers to the perception of an individual within an affiliated group, determined by how valuable one feels within that group (membership CSES), the sense of pride and value one feels toward the group (private CSES), how others evaluate the group (public CSES), and how important one’s affiliation with the group is to one’s identity or self-conception (identity CSES; Crocker and Luhtanen, 1990). This is similar to social identification. Moreover, according to the social identity theory of Tajfel and Turner (2004), after self-evaluation, one forms identification through a comparison between one’s affiliated in-group and a comparable outgroup so as to maintain a general identity. Therefore, one has a tendency to identify oneself not only through individual ability and belief in the professional responsibility to educate (teacher efficacy) but also through a sense of general satisfaction with—and pride in—the affiliated group (a product of evaluating oneself and others, which is also known as CSES). Like teacher efficacy, CSES is a variable that can influence one’s commitment to the affiliated group. Korea, a nation with a collectivistic culture, demonstrates an inclination toward collectivism based on its collective identity (Chung, 2012); in such a culture, one is more likely to agree with the organization if the relevant collectivist values are reflected in one’s self-concept. East Asian collectivist cultures create a strong tendency where individuals within a given culture identify themselves with their respective roles and positions in the family, group, workplace, and community, and they strive to achieve and maintain their personal relationships, group harmony, and common goals (Triandis, 1989; Markus and Kitayama, 1991). In fact, it has been suggested that CSES may function as a positive influence or play a mediating role in relation to organizational vocation or commitment (or conversely, job burnout) (e.g., Seol and Lim, 2013; Lee et al., 2015; Oh and Choi, 2016).

According to the JD-R model, in addition to teacher efficacy and CSES, the identification and intervention of job resources are necessary for organizational commitment to be realized to its full extent. Job resources influence organizational commitment by acting as a static function for achieving an organization’s goal to reduce the physiological and psychological losses associated with the demands of a job and to interact with job demands in a manner that stimulates personal development (Demerouti et al., 2001). Although these are the main variables that induce organizational commitment, once teacher efficacy and CSES become the sole emphases, the variables may conversely lead to job demands that degrade morale and commitment by attributing the success or failure of organizational commitment to either individual teachers or the organization. Although the job resources required by each individual may differ, promotion, reward, compensation (Eberts et al., 2002; Benevene et al., 2018), social support, autonomy, and performance feedback (Taris and Feij, 2004; Salanova et al., 2005; Bakker and Demerouti, 2007) generally lead to a synchronization process. Among these resources, social support displays realistic significance for the organization’s health. According to a World Health Organization (WHO) study of mental disorders involving subjects of various ages and occupations, among seven determinants of mental health and twenty factors presented as protective against mental disorder, social support was the only common factor (World Health Organization [WHO], 2004).

Social support is a broad term that refers to supportive behavior in different social environments (Helgeson, 2003) and the positive resources that individuals acquire through relationships. Social support, in the form of the information, evaluation, encouragement, and praise offered by others, helps individuals overcome stressful situations and reduces negative physiological or psychological reactions, thereby enhancing adaptability to the organization (Schwarzer and Knoll, 2007). For example, for the personal assistants of people living with disabilities, social support performed a protective function against job stress and acted as a motivator for their tasks (Chung et al., 2017). Additionally, for nurses, the level of job performance was high for individuals who perceived that they received social support from colleagues (AbuAlRub, 2004). In studies on teachers who educate relatively young children, similar to the participants in the current study, social support was positively linked to organizational commitment (Cho et al., 2008) and negatively linked to workload, which was related to lower levels of work- and student-related burnout (Avanzi et al., 2018) and also affected well-being (Chi et al., 2014). Social support also has a positive effect on teacher efficacy, an effect that has been observed in childcare teachers, special education school teachers, and elementary, middle, and high school teachers (Cho et al., 2008; Shen, 2009; Minghui et al., 2018). Researchers have suggested that teacher efficacy should not be accounted for as a result of an individual’s ability but ultimately rests on the teachers’ perception of positive and supportive information as provided within the social network (Cho et al., 2008; Chung, 2013).

Meanwhile, cross-cultural studies on social support have interesting findings. Unsatisfactory support was found to be associated with higher emotional exhaustion in Italian teachers compared to Swiss teachers (Fiorilli et al., 2015). In other studies, Hong Kong teachers demonstrated that their higher job satisfaction was linked to their wages and promotion, while Italian teachers demonstrated greater satisfaction from supervision, work conditions, relationships with coworkers, the network of work, and communication (Benevene et al., 2018). This can be interpreted to explain why social support plays a much more important role for Italian teachers than Swiss teachers (Avanzi et al., 2018), as Italian teachers can be more vulnerable to stress due to lower salaries and more restrictions on career advancement than teachers in other European countries (Benevene et al., 2018). In fact, the factors that influence the satisfaction of Italian teachers are highly relevant to social support. However, the relative importance of social support should not be considered only with respect to cultural differences; studies should also take into account the occupational structural aspects, in which rapid promotion and salary negotiation are not possible according to individuals’ abilities. Despite being within the same culture, Korean childcare teachers are more negatively affected by low pay and long working hours than elementary, middle, and high school teachers. As an organization is not maintained solely through one individual’s professional abilities, social support can function as a safety net (Chung, 2013) and have a positive effect on strengthening CSES and collective consciousness. Additionally, social support can promote psychological stability in the workplace by strengthening unity between members of a group (Jang, 2015). Thus, social support could improve the professional environment of Korean childcare teachers.

Social support plays a buffering role against hindrances to team cohesion and unity, while it plays a strengthening role in the promotion of healthy behavior and attitudes. Nevertheless, previous research has continued to insist on the importance of each variable and in particular, of the partial mediating or moderating effect of social support on the relationship between only two variables. Hence, this study aimed to confirm the functional point of social support in a comprehensive relationship and to determine an integrated model of this function. In other words, this study’s purpose is to examine whether social support, as a strengthening role, demonstrates a moderating effect among the relationship of three variables, resulting in a model in which CSES (mediating variable) mediates between teacher efficacy (predictor variable) and organizational commitment (outcome variable). As a basis for a more rational education policy direction that could encourage the organizational commitment of childcare teachers, this study contributes empirical evidence for when and how social support can intervene to achieve the goals of the educational organization. Based on the literature reviewed above, this study proposes the following hypotheses:

Hypothesis 1: Collective self-esteem partially mediates the relationship between teacher efficacy and organizational commitment, and teacher efficacy has a greater influence on increased organizational commitment via CSES.
Hypothesis 2: Social support moderates the direct and/or indirect associations between teacher efficacy and organizational commitment via CSES. Specifically, the direct and/or indirect associations between teacher efficacy and organizational commitment are much stronger when perceived social support is higher.

## Materials and Methods

### Participants and Procedures

This study utilized a self-reported survey of childcare teachers in D City, Korea. A public announcement for this study was made by the Graduate School of Education, which networks with each school in the area. A total of 25 nursery schools were selected (five from each region of D City), representing 20% of all the nursery schools in the city. In total, 226 surveys were collected with informed consent, and 212 completed questionnaires were included in the final analysis for a valid response rate of 93.81%. The mean age of the participants was 32.10 years (*SD* = 8.69, range = 20–53). Among all of the participants, 50.9% were single, and 49.1% were married. The average number of working hours per day were classified as less than 8 h (12.3%), 8–10 h (48.1%), 10–12 h (33.0%), and more than 12 h (6.6%).

### Measures

#### Teacher Efficacy

Teacher efficacy was measured using Shin’s 22-item teacher efficacy scale for Korean childcare teachers Shin (2004), which was based on Gibson and Dembo’s (1984) Teacher Efficacy Scale. This scale consists of two subscales, including personal efficacy (e.g., “I have sufficient practical knowledge to be effective in guiding a child.”) and general efficacy (e.g., “When a child shows a more enthusiastic attitude than usual, it is often because the teacher exerted a little extra effort.”). The participants rated their agreement with each item ranging from 1 (*strongly disagree*) to 5 (*strongly agree*). Regarding internal consistency, Shin’s scale had a Cronbach’s alpha coefficient of 0.88 in a sample of Korean childcare teachers Shin (2004), and in the current study, the Cronbach’s alpha was 0.87.

#### Collective Self-Esteem

A Korean translation of the sixteen-item CSES (Luhtanen and Crocker, 1992) was used to measure teachers’ CSES. This scale consists of four subscales: membership [e.g., “I am a worthy member of the (teachers) group I belong to.”], private [e.g., “In general, I’m glad to be a member of the (teachers) group I belong to.”], public [e.g., “In general, others respect the (teachers) group that I am a member of.”], and identity [e.g., “The (teachers) group I belong to is an important reflection of who I am.”]. Participants rated their agreement with each item ranging from 1 (*strongly disagree*) to 5 (*strongly agree*). In two samples, the Cronbach’s alpha coefficient was 0.87 for undergraduates (Kim, 1993) and 0.85 for childcare teachers (Seo, 2014), and in the current study, the Cronbach’s alpha was 0.86.

#### Social Support

The Korean version of one of the three subscales, “social support,” (K-JCQ; Eum et al., 2006) of the Job Content Questionnaire (JCQ; Karasek, 1985) was used to measure social support. This subscale is composed of eight items: four-items for coworker support such as a fellow teacher and four-items for supervisor support such as a principal or vice principal. Participants rated their agreement with each item ranging from 1 (*strongly disagree*) to 5 (*strongly agree*). In two samples, the Cronbach’s alpha coefficient was 0.71 for healthcare workers (Eum et al., 2006) and 0.94 for kindergarten teachers (Chung, 2013), and in the current study, the Cronbach’s alpha was 0.92.

#### Organizational Commitment

The Korean translation of the 21-item Organizational Commitment Questionnaire (OCQ; Allen and Meyer, 1990) was used to measure organizational commitment. This scale was modified to fit early childhood teachers (Gang, 2009), and revised to a more appropriate Korean equivalent, following Korean linguistic and psychometric expert advice (Kim and Chung, 2014). This scale consists of three subscales: affective (e.g., “I enjoy discussing my organization [kindergarten/nursery school] with people outside it.”), continuance (e.g., “It would be very hard for me to leave my kindergarten/nursery school right now, even if I wanted to.”), and normative (e.g., “If I got another offer for a better job elsewhere, I would not feel it was right to leave my kindergarten/nursery school.”). Participants rated their agreement with each item ranging from 1 (*strongly disagree*) to 5 (*strongly agree*). The K-OCQ had a Cronbach’s alpha coefficient of 0.88 in a sample of preschool teachers (Gang, 2009; Kim and Chung, 2014), and in the current study, the Cronbach’s alpha was 0.88.

### Data Analysis

At the preliminary analysis phase, descriptive statistics and correlations were calculated to discern demographic characteristics and perform a normality test on the data. Then, a Harmen’s single factor test (Podsakoff and Organ, 1986) was conducted to check for the potential problem of common method bias, resulting from the use of self-reported data. At the main analysis phase, a regression-based path analytical framework was employed, which were analyzed by means of the PROCESS macro for SPSS (Hayes, 2017). The macro uses a bootstrapping technique that is implemented for the inference of indirect associations, regardless of the underlying distribution, in order to evaluate total, direct, and indirect moderating effects. This bootstrapping technique is statistically strong and controls for Type I errors (Davison and Hinkley, 1997; Preacher et al., 2007; Hayes, 2009). The technique produced 95% bias-corrected confidence intervals for the indirect effect from 5,000 resamples of the data. In addition, the significant interactions were probed using a traditional simple slope test, the pick-a-point approach (Aiken and West, 1991), and the Johnson-Neyman technique for regions of significance (Preacher et al., 2006; Hayes and Matthes, 2009). In short, this study verified the conditional indirect effect, demonstrating that the indirect effect of the predictor (teacher efficacy) on the criterion variable (organizational commitment) through a mediator (CSES) was moderated by values of social support. Although the meta-analysis confirmed that demographic characteristics, such as gender and marital status, do not affect the organizational commitment of teachers (Çogaltay, 2015), all these analyses were conducted after controlling for demographic characteristics (i.e., age, marital status, and the average number of working hours per day) in order to better understand the relationships of the major variables.

## Results

### Preliminary Analysis

The descriptive statistics and correlations of the results can be found in Table 1. The skewness and kurtosis values of all variables were between ±1, indicating that they met the acceptable range for normality (i.e., skewness < 2, kurtosis < 7) even when they were interpreted by adding 3, which is the value of the standardized kurtosis reported in SPSS (Curran et al., 1996). Unrotated factor analysis indicated that the first principle factor explained 21.00% of the total variance. Thus, no general factor was apparent, and the common method bias is unlikely to be a serious problem in this study. In addition, teacher efficacy was significantly associated with CSES, social support, and organizational commitment, and there were moderate correlations (Cohen, 1988) among the main variables.

**Table 1:** Correlations and descriptive statistics of the main variables.

Variable	1	2	3	4	*M*	*SD*	Skewness	Kurtosis
1. Teacher efficacy	–	0.496^∗∗^	0.487^∗∗^	0.374^∗∗^	76.17	9.106	0.474	0.649
2. Collective self-esteem		–	0.423^∗∗^	0.394^∗∗^	57.09	7.787	0.800	0.147
3. Social support			–	0.334^∗∗^	30.42	5.413	-0.792	1.414
4. Organizational commitment				–	69.79	11.045	-0.023	0.596


*^∗∗^*p* < 0.01.*

### Testing for Mediation

**FIGURE 1:**
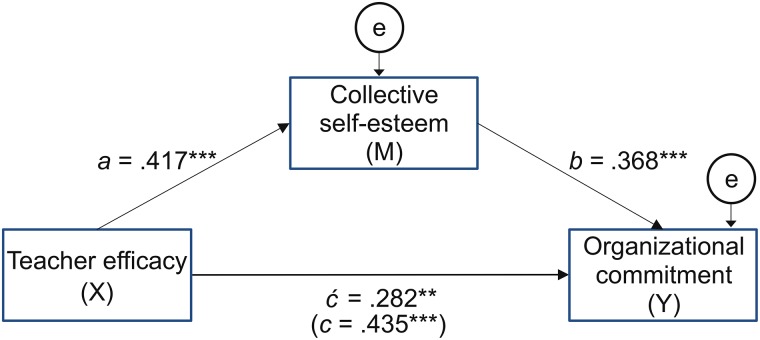
Baseline mediation model ^∗∗^p <0.01, ^∗∗∗^p <0.001.

This study tested whether CSES mediated the relation between teacher efficacy and organizational commitment, using Model 4 of the PROCESS macro (Hayes, 2017). After controlling for demographic characteristics (i.e., age, marital status, and the average number of working hours per day), teacher efficacy was significantly associated with increased CSES [*B*(a) = 0.417, *SE* = 0.053, *t* = 7.816, *p* < 0.001], and teachers’ CSES was significantly associated with increased organizational commitment [*B*(b) = 0.368, *SE* = 0.105, *t* = 3.492, *p* < 0.001], controlling for teacher efficacy (the predictor). However, the direct effect of teacher efficacy on organizational commitment, controlling for CSES (the mediator), sagged to *B*(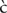) = 0.282 (*SE* = 0.092, *t* = 3.075, *p* < 0.01) in comparison with the total effect, *B*(c) = 0.435 (*SE* = 0.083, *t* = 5.268, *p* < 0.001). In addition, the indirect effect (ab = 0.153) was found to be significant (95% *CI* = [0.049, 0.287] with a bootstrapped confidence interval that did not contain zero. Therefore, CSES partially mediated the relation between teacher efficacy and organizational commitment (see Figure 1), and thus, Hypothesis 1 was supported.

### Testing for Moderated Mediation

The next procedure examined what path social support moderates in the initial mediated model. In other words, this study tested whether social support moderates the relation between teacher efficacy and organizational commitment (path 1), teacher efficacy and CSES (path 2), or CSES and organizational commitment (path 3), using Model 1 of the PROCESS macro. However, the interaction of CSES and social support was not statistically significant in path 3 (*B* = 0.028, *SE* = 0.016, *t* = 1.754, *n.s*.), and the moderating effect of social support was not found in the relationship between CSES and organizational commitment. Therefore, the last procedure, moderated mediation analysis, combined the mediation model and the moderation model into a single framework and was performed using Model 8 of the PROCESS macro (Hayes, 2017).

**FIGURE 2:**
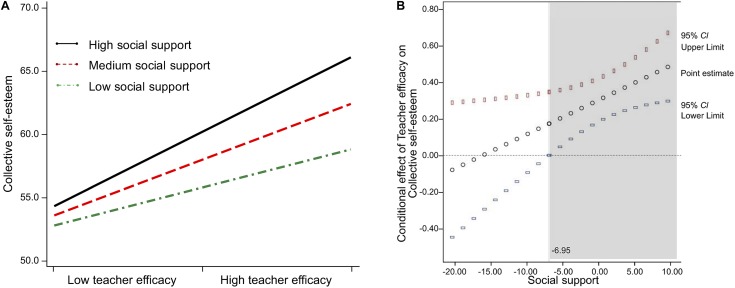
The conditional effect of teacher efficacy on collective self-esteem as a perception of social support: Simple slope, the pick-a-point approach **(A)** and Johnson-Neyman regions of significance **(B)**.

As illustrated in Table 2, the interaction of teacher efficacy and social support was significant [*B*(a_3_) = 0.018, *SE* = 0.008, *t* = 2.249, *p* < 0.05], and explained an additional 1.6% of variance in CSES [*F*(1,203) = 5.058, *p* < 0.05]. The incremental variance accounted for by the interaction term was substituted into *f*-squared, Cohen’s effect size formula, because *R*-squared change is not linearly related to effect size (Aiken and West, 1991). The *f*^2^ value is 0.025, which is larger than Cohen’s guideline threshold of 0.02 (Cohen, 1988). This means that the effects of teacher efficacy on CSES depend on the level of social support (θ_X→*M*_ = a_l_ + a_3_*W*). This is the first component of the indirect effect of teacher efficacy (*X*) on organizational commitment (*Y*) through CSES (*M*). For the first interaction pattern, simple slopes of teacher efficacy on changes in CSES changes, depending on the level of social support, were tested for low (-1 *SD* below the mean), medium (mean), and high (+1 *SD* above the mean) social support. Figure 2A illustrates that the relationship between teacher efficacy and CSES was stronger for high social support than it was for low social support. For low social support (-1 *SD* = -5.413), the effect of teacher efficacy on changes in CSES was significant (*b* = 0.197, *t* = 2.562, *p* < 0.05, 95% *CI*: [0.045, 0.347]) as this interval did not contain zero; for medium social support (mean = 0.00), the effect was significant (*b* = 0.296, *t* = 5.070, *p* < 0.001, 95% *CI*: [0.181, 0.411]); and for high social support (+1 *SD* = 5.413), the effect was significant (*b* = 0.395, *t* = 5.706, *p* < 0.001, 95% *CI*: [0.259, 0.532]). In addition, this study probed the interaction by using the Johnson-Neyman technique (Hayes and Matthes, 2009), which mathematically derives the regions of significance for the conditional effect of teacher efficacy, meaning the values within the range of the moderator in which the association between teacher efficacy and CSES is statistically different to zero. Figure 2B plots the conditional effect of teacher efficacy on CSES across the distribution of social support as well as the upper and lower bounds of a 95% confidence interval for the conditional effect. As can be seen, when social support is greater than -6.95, the effect of teacher efficacy is significantly positive and different to zero.

**Table 2:** Regression results for the moderated mediation model.

	Collective self-esteem (*M*)				Organizational commitment (*Y*)			
		
Variable		*B*	*SE*	*t*		*B*	*SE*	*t*
Teacher efficacy (*X*)	*a*_1_	0.296	0.058	5.070^∗∗∗^	* 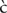 *_1_	0.194	0.094	2.062^∗^
Collective self-esteem (*M*)		–	–	–	*b*_1_	0.250	0.107	2.343^∗^
Social support (W)	*a*_2_	0.410	0.096	4.282^∗∗∗^	* 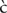 *_2_	0.469	0.152	3.086^∗∗^
Teacher efficacy × Social support (XW)	*a*_3_	0.018	0.008	2.249^∗^	* 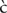 *_3_	0.043	0.013	3.417^∗∗∗^
Constant	*i*_1_	57.433	2.621	21.917^∗∗∗^	*i*_2_	55.821	7.307	7.639^∗∗∗^
	*R*^2^ = 0.359				*R*^2^ = 0.267			
	*F*(8,203) = 14.195^∗∗∗^				*F*(9,202) = 8.194^∗∗∗^			


*^∗^*p* < 0.05, ^∗∗^*p* < 0.01, ^∗∗∗^*p* < 0.001.*

The second component is the effect of CSES (*M*) on organizational commitment (*Y*), controlling teacher efficacy (*X*). This effect is not proposed as moderated (see aforementioned path 3), so this effect can be represented with a single estimate, b_1_, that teachers’ CSES was significantly associated with increased organizational commitment [*B*(b_1_) = 0.250, *SE* = 0.107, *t* = 2.343, *p* < 0.05]. Multiplying these two components yields the indirect effect of teacher efficacy (*X*) on organizational commitment (*Y*) through CSES (*M*): θ_X__→_*_M_b*_1_ = (*a*_l_ + *a*_3_*W*)*b*_l_ = (0.296 + 0.018*W*) (0.250). Given the previous evidence that the *X* → *M* path is moderated by *W*, the next step is the estimation of the conditional indirect effect for various values of *W*, along with an inferential test at those values. As a result, as the interaction between teacher efficacy and social support was linked, the indirect effect of teacher efficacy on organizational commitment through CSES depended on the level of social support. With the conditional indirect effect, low social support led to a flat increase in organizational commitment, and with higher social support, organizational commitment increased more rapidly: for low social support (-1 *SD* = -5.413), *b* = 0.049, 95% *CI*: [0.006, 0.160]; for medium social support (mean = 0.00), *b* = 0.074, 95% *CI*: [0.012, 0.190]); and for high social support (+1 *SD* = 5.413), *b* = 0.099, 95% *CI*: [0.015, 0.244].

Finally, the direct effect of teacher efficacy (*X*) on organizational commitment (*Y*) estimated how differences in *X* relate to differences in *Y*, holding constant the proposed CSES (*M*). Evidence of moderation is found in a statistically significant coefficient for the product term (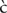
_3_ = 0.043, *SE* = 0.013, *t* = 3.417, *p* < 0.001). Thus, the direct effect of teacher efficacy influences organizational commitment independent of CSES, but is dependent on social support, which means that the direct effect is conditional (θ_X→*Y*_ = 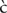
_1_ + 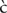
_3_*W* = 0.194 + 0.043*W*).

**FIGURE 3:**
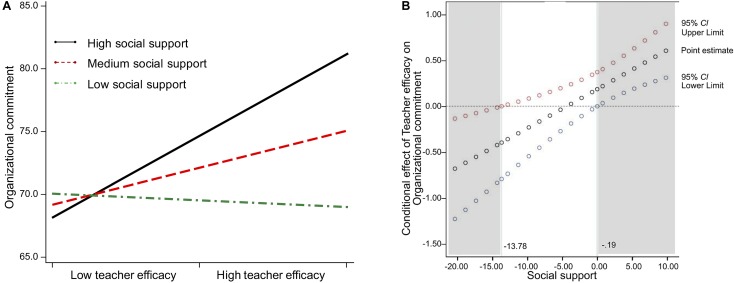
The conditional indirect effect of teacher efficacy on organizational commitment through collective self-esteem as a perception of social support: Simple slope, the pick-a-point approach **(A)** and Johnson-Neyman regions of significance **(B)**.

**FIGURE 4:**
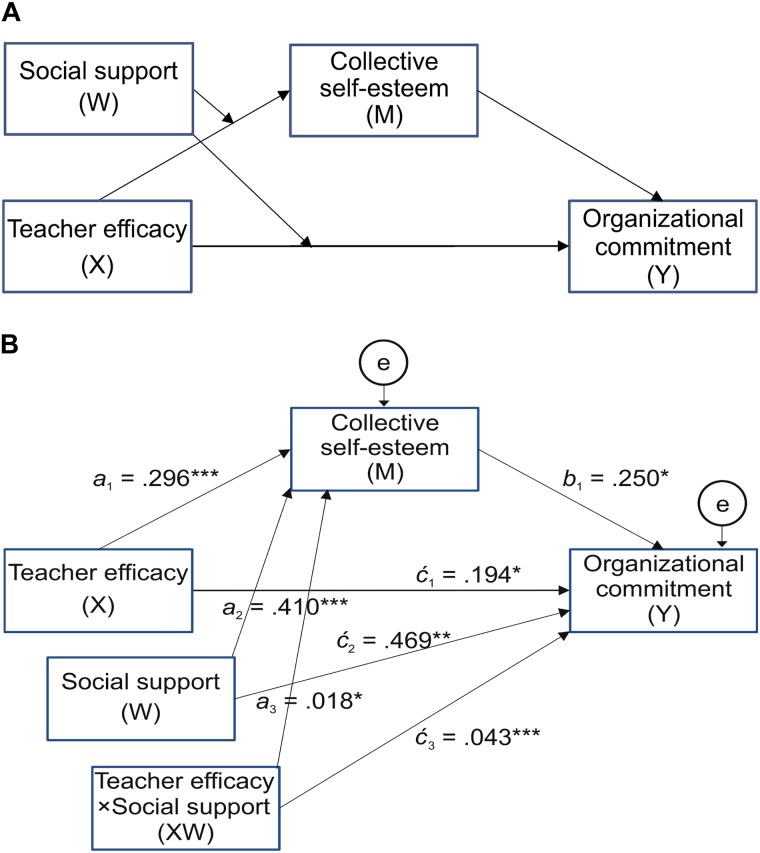
The final model: Conceptual diagram **(A)** and statistical diagram **(B)**. ^∗^p <0.05, ^∗∗^p < 0.01, ^∗∗∗^p <0.001.

As illustrated in Figure 3A, the pattern of the simple slope indicates that, among childcare teachers with less social support, the relationship is negative, as organizational commitment decreases despite the increase in teacher efficacy. The relationship flattens and then even appears to become positive as social support increases. In addition, this analysis of the region of significance found a lower bound of -13.78 and an upper bound of -0.19 (see Figure 3B). With social support greater than -0.19, the effect of teacher efficacy is statistically positive and different to zero, whereas, when social support is less than -13.78, the effect of teacher efficacy is significantly negative. Between these two values, there is no evidence of an association between teacher efficacy and organizational commitment. A 95% bootstrap confidence interval for this moderated mediation (*a*_3_*b*_l_ = 0.005) was found to be entirely above zero (0.001–0.015), meaning that CSES can be construed as a mediator of the moderating effect of teacher efficacy on organizational commitment through social support. These findings support Hypothesis 2, as illustrated in Figure 4.

## Discussion

This study investigated the dynamics of childcare teachers and organizational commitment from the perspective of the human resource management of an educational organization. The results demonstrate that the two main hypotheses were supported. First, teacher efficacy led to increased organizational commitment, mediated by CSES (Hypothesis 1). In other words, although teacher efficacy promoted organizational commitment, the increase in organizational commitment was better explained by considering the mediating variable rather than the direct effect of teacher efficacy. For studies in Korea (e.g., Seol and Lim, 2013; Lee et al., 2015), CSES has its own significance and suggests that it functions better as a mediating role when explaining organizations. This study also provides further evidence for previous studies’ proposals regarding the relationship between the three variables. Although many studies agree that teacher efficacy leads to teacher commitment, teacher efficacy should be regarded as a variable with significant influence on CSES or organizational commitment within the affiliated group. At least this is the case in East Asian cultures, which are characterized by a high level of identification with family, work, and community. This result reiterates a related study (Butler and Constantine, 2005) that states that CSES is useful when dealing with job-related variables as a group rather than as an independent individual.

Next, this study investigated the integrated role of social support within the baseline model of teacher efficacy, CSES, and organizational commitment. It was suggested that the effects of teacher efficacy on organizational commitment via CSES would be moderated through social support (Hypothesis 2), and the results supported this hypothesis. Unlike other relationships, the relationship between CSES and organizational commitment demonstrated no moderating effect of social support, which signals a need to examine when and how social support comes into effect through a more integrated relationship between the variables. As a result, perceived social support moderated the effect of teacher efficacy on CSES, and the indirect effect of teacher efficacy on organizational commitment, via CSES, relied on the level of perceived social support. In other words, the higher the perceived social support, the more teacher efficacy increases the commitment to the educational institution. This is partially attributed to higher perceived CSES and higher perceived social support from one’s principal or coworkers with regards to the teachers’ pride in their associated group. Coworker support can help increase teachers’ sense of belonging and commitment (Travers, 2017), and supervisor support can lead to an increase in organizational commitment through interventions focusing on engagement, stress, and burnout related to their work (Hakanen et al., 2006; Chung, 2013). In particular, inexperienced teachers will be able to feel more supported and then be more committed to the organization by developing expertise through the support of their superiors (Masilamani et al., 2012; Chung, 2013; Fiorilli et al., 2019). Therefore, social support exacerbates the solidification of the relationship between teacher efficacy, CSES, and organizational commitment, and could be used to induce voluntary commitment when establishing CSES in an organization, rather than when CSES is already well established. Also, as social support increased, teacher efficacy positively influenced organizational commitment regardless of perceived CSES. However, when social support was perceived to be low, organizational commitment decreased even though teacher efficacy increased. Although it may be interpreted as an exaggeration, social support needs to be provided beyond a certain level, and social support should at least play a buffering role against the negative aspects of an educational organization. This is a determinant of mental health and a protective factor against mental disorders, which is in line with the findings of the WHO study on social support involving various occupations (2004). Due to a recent series of child abuse scandals in nursery schools, Korean policy on childcare has started to focus more on restrictions and monitoring in childcare, including intensive inspections of nursery schools and punishment for teachers. However, strengthening the management is not the only solution. This study suggests that melding the social support system for childcare teachers with childcare policy as well as improvements to the objective environment (i.e., buildings and other facilities) will help teachers engage students and their employer organizations, thus preventing high levels of deviation. Policy aims that provide greater social support will improve the quality of nursery school education and the quality of educator experience.

Until now, this article has emphasized the positive effect of job resources, which alleviate tension and improve the performance of organization members, stressing the view that teachers’ growth may be enhanced. For example, the research examined the types of job resources, including compensation, performance feedback, and social support, and explored the availability and effect of job resources. However, the time has come not only to provide job resources but to understand how and when each job resource should be implemented to demonstrate the targeted effect and to understand the period of efficient implementation. The findings of this study extend JD-R model-related research by including a mediating mechanism as illustrated in Figure 4, in which the interaction between teacher efficacy and social support not only positively affects CSES, but also organizational commitment.

It would be meaningful to expand the social support scope covered by this study, as it identified the role and timing of social support. In the present study, social support was defined as consisting of coworker support, which was in the form of similarity, intimacy, and cooperation, and supervisor support, which played a relatively facilitative role in building professionalism. Although support itself is a very broad and inclusive term, social support should at least include human and relational factors. In addition, both schools and families are important for teachers’ lives in Korea, where it is essential to have a sense of belonging. For teachers, events at schools can affect home life, and events at home can affect school life; thus, these factors have a reciprocal shared structure. Therefore, social support can be extended to include internal and external support related to families, society, culture, and relationships. Internal support is provided by professional personnel such as peers, seniors, mentors, head teachers, and psychologists, while external support can be obtained from private networks such as family, friends, and partners (Fiorilli et al., 2017). Fiorilli et al. (2019) categorized social support for teachers into family and non-family support and reported that the family is the most important source of support in the Italian family-centered culture. This can be expressed differently as emotional support from family and problem-centered support from non-family in order to further highlight their roles (Hargreaves, 2001; Halbesleben et al., 2010; Fiorilli et al., 2019). Despite Korea’s family-centered culture (Lee, 2014), there is an expectation of sacrifice and support of family, as Korea is also a work-oriented society, with the second-longest working hours in 2016 and the third-longest in 2017 among member states of the Organization for Economic Cooperation and Development (Korean Statistical Information Service, 2018). It is also true that non-family support is important owing to the emphasis on school relations, regionalism, the apprentice system, and the Korean cultural concept of “Jeong (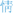)” (Chung, 2012). In these complex Korean contexts, the variables of this study have been strictly limited to the generalized educational fields in which childcare teachers work; however, it would be worth examining social support beyond this area in the future.

With this in mind, the limitations and related directions for future study are as follows. First, in terms of the effectiveness of job resources, many studies besides the current one emphasize the importance of social support. However, as mentioned earlier, this study only included the support of supervisors (principals and vice principals) and coworkers, whose roles constitute the organization. Future research should examine the scope and quality of social support and the social support network, and it should determine the measures and structures necessary to maximize the effectiveness of support by conducting qualitative analysis through interviews, without relying on the existing criteria. In addition, a comparative cross-cultural approach to examine the extent of validity in which social support functions may be proposed would also be useful. Second, in terms of the overall manner in which the impact of job resources was demonstrated, this study presented CSES as a mediating variable. This is due to the ordinary view that the perception and evaluation of job groups are important for understanding job-related variables, which, like organizational commitment, many members of the group share in common (Butler and Constantine, 2005). This also may simply be a characteristic of Korea’s collectivistic culture. However, some studies demonstrate that European Americans pursue greater social support and perceive a higher degree of utility than East Asians (e.g., Taylor et al., 2004). Therefore, in order to confirm whether this is a general path, future research needs to conduct comparative research with different domestic and foreign samples repeatedly. Third, this study considered other variables as antecedents to organizational commitment from the perspective of the human resource management of childcare teachers. Prior studies have demonstrated that organizational commitment is a product of results, as measured by performance and productivity, in addition to affective aspects (Chung, 2015), personal qualities, and how an individual is defined by being a member of an organization, all of which may be precursors to the development of a positive attitude toward and commitment to an organization (Meyer et al., 2004; Stinglhamber et al., 2015). However, the cross-sectional data of this study have limitations, making it difficult to prove the causality of the variables. A time variable is also necessary to examine not only changes in performance outcome but also whether social support, which has followed the path outlined in this study, has been practically implemented in organizational commitment. Longitudinal designs and cross-lagged panel design between measurement times may be considered in any expansion of the present work.

## Ethics Statement

This study was carried out in accordance with the recommendations of the standard operating guideline of Joongbu University Institutional Reviews Board (Approval No. JIRB-201509160101151030) with written informed consent from all subjects. All subjects gave written informed consent in accordance with the Declaration of Helsinki. The protocol was approved by the Joongbu University Institutional Reviews Board.

## Author Contributions

M-SC contributed to the conception or design, acquisition, analysis, or interpretation of data, drafted the work or revised it critically for important intellectual content, approved the final version of the manuscript to be published, and agreed to be accountable for all aspects of the work.

## Conflict of Interest Statement

The author declares that the research was conducted in the absence of any commercial or financial relationships that could be construed as a potential conflict of interest.

## References

[B1] Abbey D. E. Esposito J. P. (1985). Social support and principal leadership style: a means to reduce teacher stress. *Education* 105 327–332.

[B2] AbuAlRub R. F. (2004). Job stress, job performance, and social support among hospital nurses. *J. Nurs. Scholarsh.* 36 73–78. 10.1111/j.1547-5069.2004.04016.x 15098422

[B3] Aiken L. S. West S. G. (1991). *Multiple Regression: Testing and Interpreting Interactions.* Newbury Park, CA: Sage.

[B4] Allen N. J. Meyer J. P. (1990). The measurement and antecedents of affective, continuance and normative commitment to the organization. *J. Occup. Organ. Psychol.* 63 1–18. 10.1111/j.2044-8325.1990.tb00506.x

[B5] Ashton P. T. (1985). “Motivation and the teacher’s sense of efficacy,” in *Research on Motivation in Education* Vol. 2 eds Ames C. Ames R. (Orlando, FL: Academic Press), 141–174.

[B6] Avanzi L. Fraccaroli F. Castelli L. Marcionetti J. Crescentini A. Balducci C. (2018). How to mobilize social support against workload and burnout: the role of organizational identification. *Teach. Teach. Educ.* 69 154–167. 10.1016/j.tate.2017.10.001

[B7] Bakker A. B. Demerouti E. (2007). The Job Demands-Resources model: state of the art. *J. Manag. Psychol.* 22 309–328. 10.1108/02683940710733115

[B8] Benevene P. Wong Y. H. P. Fiorilli C. Stasio S. D. (2018). A cross-national comparison on subjective well-being of kindergarten teachers: Hong Kong and Italy. *Front. Psychol.* 9:2626. 10.3389/fpsyg.2018.02626 30631298 PMC6315170

[B9] Bogler R. Somech A. (2004). Influence of teacher empowerment on teachers’ organizational commitment, professional commitment, and organizational citizenship behavior in schools. *Teach. Teach. Educ.* 20 277–289. 10.1016/j.tate.2004.02.003

[B10] Broudy H. S. (1984). “The university and the preparation of teachers,” in *Advances in Teacher Education* Vol. 1 eds Katz L. G. Raths J. D. (Norwood, NJ: Ablex Publishing Corporation), 1–8.

[B11] Butler S. K. Constantine M. G. (2005). Collective self-esteem and burnout in professional school counselors. *Prof. Sch. Couns.* 9 55–62. 10.1177/2156759X0500900107

[B12] Chi H. Yeh H. Wu S. F. (2014). How well-being mediates the relationship between social support and teaching effectiveness. *J. Educ. Learn.* 3 117–130. 10.5539/jel.v3n4p117

[B13] Cho S.-J. Moon S.-B. Min H.-Y. (2008). The influence of the self-efficacy and the social support on the organizational commitment of kindergarten and child care teachers. *J. Korean Home Manag. Assoc.* 26 25–32.

[B14] ChungM.-S. (2012). Moderating effects of empathy on the relationship between Cheong ( 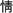 ) and forgiveness. *Korean J. Rehabil. Psychol.* 19 407–421.

[B15] Chung M.-S. (2013). Relationship between kindergarten teachers’ burnout and turnover intention: the moderating effect of social support. *J. Humanit.* 33 183–204.

[B16] Chung M.-S. (2015). Mediating effects of job stress and moderating effects of autonomy on the relationship between emotional labor and organizational commitment. *Korea J. Couns.* 16 121–138. 10.15703/kjc.16.3.201506.121

[B17] Chung M.-S. Lee K.-J. Han G.-H. (2017). Effects of social support and self-regulation on job stress: focused on personal assistants for the disabled. *J. Korea Acad. Industr. Coop. Soc.* 18 265–273. 10.5762/KAIS.2017.18.10.265

[B18] Çogaltay N. (2015). Organizational commitment of teachers: a meta-analysis study for the effect of gender and marital status in Turkey. *Educ. Sci. Theory Pract.* 15 911–924. 10.12738/estp.2015.4.2755

[B19] Cohen J. (1988). *Statistical Power Analysis for the Behavioral Sciences*, 2nd Edn Hillsdale, NJ: Lawrence Erlbaum Associates.

[B20] Crocker J. Luhtanen R. (1990). Collective self-esteem and ingroup bias. *J. Pers. Soc. Psychol.* 58 60–67. 10.1037/0022-3514.58.1.60

[B21] Curran P. J. West S. G. Finch J. F. (1996). The robustness of test statistics to nonnormality and specification error in confirmatory factor analysis. *Psychol. Methods* 1 16–29. 10.1037/1082-989X.1.1.16

[B22] Davison A. C. Hinkley D. V. (1997). *Bootstrap Methods and their Application.* Cambridge: Cambridge University Press.

[B23] Demerouti E. Bakker A. B. Nachreiner F. Schaufeli W. B. (2001). The job demands-resources model of burnout. *J. Appl. Psychol.* 86 499–512. 10.1037/0021-9010.86.3.499 11419809

[B24] Dunham R. B. Grube J. A. Castañeda M. B. (1994). Organizational commitment: the utility of an integrative definition. *J. Appl. Psychol.* 79 370–380. 10.1037/0021-9010.79.3.370

[B25] Eberts R. Hollenbeck K. Stone J. (2002). Teacher performance incentives and student outcomes. *J. Hum. Resour.* 37 913–927. 10.2307/3069621

[B26] Eum K.-D. Li J. Jhun H.-J. Park J.-T. Tak S.-W. Karasek R. (2006). Psychometric properties of the Korean version of the job content questionnaire: data from health care workers. *Int. Arch. Occup. Environ. Health* 80 497–504. 10.1007/s00420-006-0156-x 17072637

[B27] Fiorilli C. Albanese O. Gabola P. Pepe A. (2017). Teachers’ emotional competence and social support: assessing the mediating role of teacher burnout. *Scand. J. Educ. Res.* 61 127–138. 10.1080/00313831.2015.1119722

[B28] Fiorilli C. Gabola P. Pepe A. Meylan N. Curchod-Ruedi D. Albanese O. (2015). The effect of teachers’ emotional intensity and social support on burnout syndrome. A comparison between Italy and Switzerland. *Eur. Rev. Appl. Psychol.* 65 275–283. 10.1016/j.erap.2015.10.003

[B29] Fiorilli C. Schneider B. Buonomo I. Romano L. (2019). Family and nonfamily support in relation to burnout and work engagement among Italian teachers. *Psychol. Sch.* 56 781–791. 10.1002/pits.22235

[B30] Gang J. Y. (2009). *A Study on the Relation among Empowerment, Job Satisfaction and Organizational Commitment of Kindergarten Teachers.* Master’s thesis, Korea National University of Education, Cheongju.

[B31] Gibson S. Dembo M. H. (1984). Teacher efficacy: a construct validation. *J. Educ. Psychol.* 76 569–582. 10.1037//0022-0663.76.4.569

[B32] Goddard R. D. (2001). Collective efficacy: a neglected construct in the study of schools and student achievement. *J. Educ. Psychol.* 93 467–476. 10.1037/0022-0663.93.3.467

[B33] Hakanen J. J. Bakker A. B. Schaufeli W. B. (2006). Burnout and work engagement among teachers. *J. Sch. Psychol.* 43 495–513. 10.1016/j.jsp.2005.11.001

[B34] Halbesleben J. R. B. Zellars K. L. Carlson D. S. Perrewé P. L. Rotondo D. (2010). The moderating effect of work-linked couple relationships and work-family integration on the spouse instrumental support-emotional exhaustion relationship. *J. Occup. Health Psychol.* 15 371–387. 10.1037/a0020521 21058852

[B35] Hargreaves A. (2001). The emotional geographies of teachers’ relations with colleagues. *Int. J. Educ. Res.* 35 503–527. 10.1016/S0883-0355(02)00006-X 27681147

[B36] Hayes A. F. (2009). Beyond Baron and Kenny: statistical mediation analysis in the new millennium. *Commun. Monogr.* 76 408–420. 10.1080/03637750903310360

[B37] Hayes A. F. (2017). *Introduction to Mediation, Moderation, and Conditional Process Analysis: A Regression-Based Approach*, 2nd Edn New York, NY: Guilford Press.

[B38] Hayes A. F. Matthes J. (2009). Computational procedures for probing interactions in OLS and logistic regression: SPSS and SAS implementations. *Behav. Res. Methods* 41 924–936. 10.3758/BRM.41.3.924 19587209

[B39] Helgeson V. S. (2003). Social support and quality of life. *Qual. Life Res.* 12 25–31. 10.1023/A:1023509117524 12803308

[B40] Jang J.-H. (2015). Mediating effects of collective self-esteem on the relationships between social support and organizational citizenship behavior. *J. Ind. Econ. Bus.* 28 2129–2153.

[B41] Karasek R. A. (1985). *Job Content Questionnaire and User’s Guide.* Lowell, MA: University of Massachusetts Lowell.

[B42] Kim A. Kim M.-J. (2004). Validation of teacher-efficacy scale. *Korean J. Educ. Psychol.* 18 37–58. 14714928

[B43] Kim H.-S. (1993). Korean collective self-esteem scale. *Korean Psychol. Assoc. Annu. Conf.* 1993 289–301.

[B44] Kim Y.-S. Chung M.-S. (2014). Effects of early childhood teachers’ job stress on organizational commitment: the mediating role of teaching efficacy belief. *J. Korea Acad. Industr. Coop. Soc.* 15 1424–1435. 10.5762/KAIS.2014.15.3.1424

[B45] Korea Research Institute for Vocational Education and Training (2013). The status of the job in emotional labor. *Krivet Issue Brief.* 26 1–4.24143851

[B46] Korean Statistical Information Service (2018). *Average Annual Hours Actually Worked Per Worker (OECD).* Available at: http://kosis.kr/statHtml/statHtml.do?orgId=101&tblId=DT_2KAA314_OECD (accessed March 17, 2019).

[B47] Kushman J. W. (1992). The organizational dynamics of teacher workplace commitment: a study of urban elementary and middle schools. *Educ. Admin. Q.* 28 5–42. 10.1177/0013161X92028001002

[B48] Lee J. Lee H.-R. Chung C.-H. (2015). The mediating effect of collective self-esteem on the relationship between emotional labor and burnout of early childhood teachers. *J. Child. Lit. Educ.* 16 617–636.

[B49] Lee S. M. (2014). Korean families between familism and individualization: focused on the difference of familism and gender of the latent structure of intergenerational relations. *Fam. Cult.* 26 1–36. 10.21478/family.26.3.201409.001

[B50] Lee Y.-S. Lee H.-S. (2012). The relationship among school organizational health, personal teacher efficacy, collective teacher efficacy, and teaching professionalism. *J. Korean Teach. Educ.* 29 541–564. 10.24211/tjkte.2012.29.4.541

[B51] Lin Y.-C. Magnuson K. A. (2018). Classroom quality and children’s academic skills in child care centers: understanding the role of teacher qualifications. *Early Child. Res. Q.* 42 215–227. 10.1016/j.ecresq.2017.10.003

[B52] Luhtanen R. Crocker J. (1992). A collective self-esteem scale: self-evaluation of one’s social identity. *Pers. Soc. Psychol. Bull.* 18 302–318. 10.1177/0146167292183006

[B53] Ma S.-J. (2004). *The Relationship between Value Orientations towards Vocational Education and Organizational Commitment of Vocational High School Teachers.* Doctoral dissertation, Seoul National University, Seoul.

[B54] Markus H. R. Kitayama S. (1991). Culture and the self: implications for cognition, emotion, and motivation. *Psychol. Rev.* 98 224–253. 10.1037/0033-295X.98.2.224

[B55] Masilamani R. Darus A. Ting A. S. Ali R. Mahmud A. B. A. David K. (2012). Salivary biomarkers of stress among teachers in an urban setting. *Asia Pac. J. Public Health* 24 278–287. 10.1177/1010539510393725 21385771

[B56] Meyer J. P. Allen N. J. (1991). A three-component conceptualization of organizational commitment. *Hum. Resour. Manage. Rev.* 1 61–89. 10.1016/1053-4822(91)90011-Z

[B57] Meyer J. P. Becker T. E. Vandenberghe C. (2004). Employee commitment and motivation: a conceptual analysis and integrative model. *J. Appl. Psychol.* 89 991–1007. 10.1037/0021-9010.89.6.991 15584837

[B58] Meyer J. P. Herscovitch L. (2001). Commitment in the workplace: toward a general model. *Hum. Resour. Manage. Rev.* 11 299–326. 10.1016/S1053-4822(00)00053-X

[B59] Minghui L. Lei H. Xiaomeng C. Potměšilc M. (2018). Teacher efficacy, work engagement, and social support among Chinese special education school teachers. *Front. Psychol.* 9:648. 10.3389/fpsyg.2018.00648 29867634 PMC5949842

[B60] Moore W. Esselman M. (1992). “Teacher efficacy, power, school climate, and achievement: a desegregating district’s experience,” in *Paper Presented at the Annual Meeting of the American Educational Research Association*, San Francisco, CA.

[B61] Mowday R. T. Steers R. M. Porter L. W. (1979). The measurement of organizational commitment. *J. Vocat. Behav.* 14 224–247. 10.1016/0001-8791(79)90072-1

[B62] Oh S.-J. Choi J.-A. (2016). Effects of childcare teachers’ collective self-esteem on burnout. *J. Parent Educ.* 8 185–199.

[B63] Podsakoff P. M. Organ D. W. (1986). Self-reports in organizational research: problems and prospects. *J. Manage.* 12 531–544. 10.1177/014920638601200408 8452065

[B64] Preacher K. J. Curran P. J. Bauer D. J. (2006). Computational tools for probing interactions in multiple linear regression, multilevel modeling, and latent curve analysis. *J. Educ. Behav. Stat.* 31 437–448. 10.3102/10769986031004437

[B65] Preacher K. J. Rucker D. D. Hayes A. F. (2007). Addressing moderated mediation hypotheses: theory, methods, and prescriptions. *Multivar. Behav. Res.* 42 185–227. 10.1080/00273170701341316 26821081

[B66] Riggs I. M. Enochs L. G. (1990). Toward the development of an elementary teacher’s science teaching efficacy belief instrument. *Sci. Educ.* 74 625–637. 10.1002/sce.3730740605

[B67] Robbins S. P. (2001). *Organizational Behavior: Concepts, Controversies, and Applications*, 9th Edn Englewood Cliffs, NJ: Prentice-Hall.

[B68] Ross J. A. (1992). Teacher efficacy and the effects of coaching on student achievement. *Can. J. Educ.* 17 51–65. 10.2307/1495395 28232767

[B69] Saka M. Bayram H. Kabapinar F. (2016). The teaching processes of prospective science teachers with different levels of science-teaching self-efficacy belief. *Educ. Sci. Theory Pract.* 16 915–941. 10.12738/estp.2016.3.0012

[B70] Salanova M. Agut S. Peiró J. M. (2005). Linking organizational resources and work engagement to employee performance and customer loyalty: the mediation of service climate. *J. Appl. Psychol.* 90 1217–1227. 10.1037/0021-9010.90.6.1217 16316275

[B71] Schwarzer R. Knoll N. (2007). Functional roles of social support within the stress and coping process: a theoretical and empirical overview. *Int. J. Psychol.* 42 243–252. 10.1080/00207590701396641

[B72] Seo H. S. (2014). *The Influence of Teacher Efficacy and Collective Self-Esteem on the Psychological Burnout of Nursery Teachers.* Master’s thesis, Pukyong National University, Busan.

[B73] Seol K. O. Lim J. I. (2013). Collective self-esteem, calling, and burnout among youth companions. *Korean J. Couns. Psychother.* 25 187–201.

[B74] Shen Y. E. (2009). Relationships between self-efficacy, social support, and stress coping strategies in Chinese primary and secondary school teachers. *Stress Health* 25 129–138. 10.1002/smi.1229

[B75] Shin H. Y. (2004). *Effects of Teachers’ Job Stress and Belief of Efficacy on the Quality of Teachers’ Interaction Behaviors in Child Care.* Doctoral dissertation, Yonsei University, Seoul.

[B76] Stinglhamber F. Marique G. Caesens G. Desmette D. Hansez I. Hanin D. (2015). Employees’ organizational identification and affective organizational commitment: an integrative approach. *PLoS One* 10:e0123955. 10.1371/journal.pone.0123955 25875086 PMC4395289

[B77] Tajfel H. Turner J. C. (2004). “The social identity theory of intergroup behavior,” in *Political Psychology: Key Readings*, eds Jost J. T. Sidanius J. (New York, NY: Psychology Press), 276–293. 10.4324/9780203505984-16

[B78] Taris T. W. Feij J. A. (2004). Learning and strain among newcomers: a three-wave study on the effects of job demands and job control. *J. Psychol.* 138 543–563. 10.3200/JRLP.138.6.543-563 15612610

[B79] Taylor S. E. Sherman D. K. Kim H. S. Jarcho J. Takagi K. Dunagan M. S. (2004). Culture and social support: who seeks it and why? *J. Pers. Soc. Psychol.* 87 354–362. 10.1037/0022-3514.87.3.354 15382985

[B80] Travers C. (2017). “Current knowledge on the nature, prevalence, sources and potential impact of teacher stress,” in *Educator Stress: An Occupational Health Perspective*, eds McIntyre T. M. McIntyre S. E. Francis D. J. (Cham: Springer), 23–54. 10.1007/978-3-319-53053-6_2

[B81] Triandis H. C. (1989). The self and social behavior in differing cultural contexts. *Psychol. Rev.* 96 506–520. 10.1037/0033-295X.96.3.506

[B82] Tschannen-Moran M. Hoy A. W. (2001). Teacher efficacy: capturing an elusive construct. *Teach. Teach. Educ.* 17 783–805. 10.1016/S0742-051X(01)00036-1

[B83] Tschannen-Moran M. Woolfolk Hoy A. Hoy W. K. (1998). Teacher efficacy: its meaning and measure. *Rev. Educ. Res.* 68 202–248. 10.2307/1170754 30462713

[B84] World Health Organization [WHO] (2004). *Prevention of Mental Disorders: Effective Interventions and Policy Options: Summary Report.* Geneva: WHO.

